# Todd on the Nervous System; Jameson on Epidemic Cholera; Barton on the Cause and Prevention of Yellow Fever

**Published:** 1855-12

**Authors:** 


Art. XI.—Todd *on the Nervous System;* Jameson *on Epidemic Cholera;* Barton *on the Cause and Prevention of Yellow Fever.*

Restricted now to a few lines of space, all that we can do at present is to acknowledge the receipt of these works—the first from the pen of Dr. Robert Bently Todd, of London, the second the production of an old and esteemed American physician, now no more, and the third the fruit of the observation of one who has been long familiar with the malady of which he writes, and whose voice on the subject is entitled to be heard. In all there is much matter to interest and instruct the medical reader.

